# COVID-19 subphenotypes at hospital admission are associated with mortality: a cross-sectional study

**DOI:** 10.1080/07853890.2022.2148733

**Published:** 2022-11-29

**Authors:** Kathryn Dubowski, Giovanna T. Braganza, Anne Bozack, Elena Colicino, Nicholas DeFelice, Laura McGuinn, Duncan Maru, Alison G. Lee

**Affiliations:** a Division of Pulmonary, Critical Care and Sleep Medicine, Icahn School of Medicine at Mount Sinai, New York, NY, USA; b School of Public Health, State University of New York, Downstate Health Sciences University, Brooklyn, NY, USA; c School of Public Health, Environmental Health Sciences, University of California Berkeley, Berkeley, CA, USA; d Environmental Medicine and Public Health, Icahn School of Medicine at Mount Sinai, New York, NY, USA; e Department of Global Health, Icahn School of Medicine at Mount Sinai, New York, NY, USA

**Keywords:** SARS CoV-2 infection, COVID-19, subphenotypes, mortality

## Abstract

**Background:**

We have an incomplete understanding of COVID-19 characteristics at hospital presentation and whether underlying subphenotypes are associated with clinical outcomes and therapeutic responses.

**Methods:**

For this cross-sectional study, we extracted electronic health data from adults hospitalized between 1 March and 30 August 2020 with a PCR-confirmed diagnosis of COVID-19 at five New York City Hospitals. We obtained clinical and laboratory data from the first 24 h of the patient’s hospitalization. Treatment with tocilizumab and convalescent plasma was assessed over hospitalization. The primary outcome was mortality; secondary outcomes included intubation, intensive care unit (ICU) admission and length of stay (LOS). First, we employed latent class analysis (LCA) to identify COVID-19 subphenotypes on admission without consideration of outcomes and assigned each patient to a subphenotype. We then performed robust Poisson regression to examine associations between COVID-19 subphenotype assignment and outcome. We explored whether the COVID-19 subphenotypes had a differential response to tocilizumab and convalescent plasma therapies.

**Results:**

A total of 4620 patients were included. LCA identified six subphenotypes, which were distinct by level of inflammation, clinical and laboratory derangements and ranged from a hypoinflammatory subphenotype with the fewest derangements to a hyperinflammatory with multiorgan dysfunction subphenotypes. Multivariable regression analyses found differences in risk for mortality, intubation, ICU admission and LOS, as compared to the hypoinflammatory subphenotype. For example, in multivariable analyses the moderate inflammation with fever subphenotype had 3.29 times the risk of mortality (95% CI 2.05, 5.28), while the hyperinflammatory with multiorgan failure subphenotype had 17.87 times the risk of mortality (95% CI 11.56, 27.63), as compared to the hypoinflammatory subphenotype. Exploratory analyses suggested that subphenotypes may differential respond to convalescent plasma or tocilizumab therapy.

**Conclusion:**

COVID-19 subphenotype at hospital admission may predict risk for mortality, ICU admission and intubation and differential response to treatment.

**KEY MESSAGE**
- This cross-sectional study of COVID patients admitted to the Mount Sinai Health System, identified six distinct COVID subphenotypes on admission. Subphenotypes correlated with ICU admission, intubation, mortality and differential response to treatment.

## Introduction

The Severe Acute Respiratory Syndrome Coronavirus 2 (SARS CoV2) pandemic has resulted in significant morbidity and mortality [1,2]. In the United States, there were an estimated 83.1 million total infections from March through December 2020 [3]. Of those infected, the disease burden ranged from asymptomatic to critical illness, and the mortality among hospitalized patients in the US was estimated to be 21% [4]. A number of studies have identified early clinical markers of severe coronavirus disease 2019 (COVID-19), such as fever, hyperglycemia, and elevated inflammatory markers that may be associated with worse outcomes [5–9]. Recent studies have shifted focus to COVID-19 heterogeneity [10,11]. We posit that COVID-19 heterogeneity at the time of hospital admission represents underlying subphenotypes with different natural histories, clinical and biological characteristics, outcomes and, possibly, responses to treatment [12–14]. Better characterizations of COVID-19 subphenotypes and their associations with outcomes could inform treatment options. Furthermore, secondary analyses of completed trials using COVID-19 subphenotypes may explain variable responses to therapeutics and improve targeted therapy.

It is now understood that syndromes of critical illness, such as acute respiratory disease syndrome (ARDS) and sepsis, both seen in severe COVID-19, are not singular in presentation but rather are composed of multiple underlying subphenotypes with differing associated morbidity and mortality risk. Secondary analyses of randomized controlled trials of ARDS employed latent class analysis and consistently identified hyperinflammatory and hypoinflammatory subphenotypes where the hyperinflammatory subphenotype was associated with higher risk of mortality [13,15–17]. These subphenotypes may differentially respond to therapy, although evidence is mixed [13,15,16]. Within the COVID-19 framework, published studies have evaluated whether individual measures such as oxygen saturation, creatinine, D-dimer or C-reactive protein (CRP) predict disease severity. Machine learning approaches have been applied to understand risk for COVID-19 mortality and critical illness however these approaches are limited by data missingness and sample size requirements [18–24]. More recent studies have examined COVID-19 subphenotypes at the time of ICU admission when the disease is advanced and successful interventions may be limited [25,26]. Moreover, randomized controlled trials of therapeutics recruit COVID-19 patients broadly and do not enrich for subphenotypes that may be more likely to respond to that therapeutic [27–29]. More research is needed to identify subphenotypes of disease severity on hospital presentation and assist with clinical risk stratification and treatment algorithms [30,31].

To address this knowledge gap, we conducted a retrospective analysis to identify COVID-19 subphenotypes on admission, examine whether identified subphenotypes were associated with COVID-19 outcomes, and explore differential response to therapeutics. Specifically, we leveraged electronic health record data from inpatient COVID-19 encounters within five New York City (NYC) hospitals during the Spring through Summer 2020 surge. Our primary COVID-19 clinical outcome was mortality; secondary outcomes included intensive care unit (ICU) admission, intubation and length of stay (LOS).

## Methods

### Study participants

We extracted electronic health data from all persons hospitalized with a PCR-confirmed diagnosis of COVID-19 at five Mount Sinai Hospital System Hospitals including Mount Sinai Brooklyn, Mount Sinai Queens, the Mount Sinai Hospital, Mount Sinai Morningside and Mount Sinai West. Specifically, we included patients who were aged 18 years or older, admitted between 1 March 2020 and 30 August 2020, and had a positive SARS Cov2 PCR nasal swab within 7 days of admission.

### Ethics

The study was approved by the Institutional Review Board at the Icahn School of Medicine at Mount Sinai (20-00547).

### Electronic health record data

Electronic health record data were collected from the first COVID-19 encounter through 30 August 2020 thereby capturing complete hospital admissions and outcomes over the NYC Spring 2020 COVID-19 surge. Data were obtained from the Mount Sinai Data Warehouse COVID-19 Data Sets, which obtained data from Mount Sinai’s Caboodle, Clarity and Enterprise Data Warehouse databases. The Mount Sinai Hospitals are located in the NYC boroughs of Manhattan [Mount Sinai Hospital (1141-bed), Mount Sinai Morningside (495-bed), and Mount Sinai West (514-bed)], Brooklyn [Mount Sinai Brooklyn (212-bed)] and Queens [Mount Sinai Queens (235-bed)].

For each patient encounter, we extracted date of admission, hospital, sex, age, self-reported race/ethnicity, insurance provider and date of SARS-CoV-2 PCR test. For patients with multiple encounters, data collected at the first encounter that met inclusion criteria were used in analyses. Information on medical comorbidities were extracted from the electronic health record using international classification of disease 10 (ICD 10) codes. Comorbidities were grouped into organ-specific categories. Persons with history of asthma, chronic obstructive pulmonary disease (COPD) and/or obstructive sleep apnea (OSA) were categorized as having pulmonary disease. Persons with history of hypertension (HTN), coronary artery disease (CAD), congestive heart failure (CHF) and/or myocardial infarction (MI) were categorized as having cardiovascular diseases. Persons were also categorized as having a history of cancer or obesity (as measured by ICD 10 code, or a calculated BMI >30). The number of organ-specific comorbidities were then summed.

We obtained clinical and laboratory data from the first 24 h of the patient’s first hospitalization that met inclusion criteria. Only variables with data available from at least 60% of participants or variables with a strong biological basis based on prior studies [erythrocyte sedimentation rate (ESR), interleukin 6 (IL-6), interleukin 1 beta (IL-1B)] were included. For variables with repeated observations, we identified the worst value recorded within 24 h of admission. Clinical variables included lowest oxygen saturation, lowest systolic and diastolic blood pressure, highest heart rate, and highest temperature within the first 24 h. Laboratory variables examined included inflammatory markers [C-reactive protein (CRP), ESR, IL-6, IL-1B, lactate dehydrogenase (LDH), procalcitonin, ferritin]; hematologic markers [white blood cell (WBC), hemoglobin, platelets, d-dimer, fibrinogen, prothrombin time (PT), partial thromboplastin time (PTT)); cardiac markers (troponin, brain natriuretic peptide (BNP)]; and renal and hepatic markers (alanine transaminase (ALT), aspartate aminotransferase (AST), albumin, total bilirubin, sodium, potassium, calcium, bicarbonate, blood urea nitrogen (BUN), creatinine, anion gap, glucose. Laboratory values above the laboratory-defined limit of detection were assigned the value at the limit of detection.

Patient outcomes were assessed across all hospital encounters. The primary outcome was mortality; secondary outcomes included intubation and admission to the intensive care unit (ICU). Specifically, patients listed as ‘expired’ or ‘deceased’ as per Epic discharge disposition were classified as deceased. Electronic health record mortality data also included health-record linked post-discharge deaths. Patients assigned to an ICU bed at any point of any hospitalization were classified as having an ICU admission. Patients recorded as having a surgical intubation, non-surgical airway intubation or endotracheal intubation during any encounter were classified as being intubated. Amongst survivors, we also determined length of stay (LOS) of the initial hospitalization. Data regarding COVID-19 therapeutics, including tocilizumab and convalescent plasma, administered while hospitalized were also collected across all hospital encounters.

### Covariates

We extracted the hospital in which patients were hospitalized (categorical variable), self-identified race/ethnicity (categorical variable: White, Black, Hispanic, Asian, Other), insurance provider (categorical variable: Private/Medicare, Medicaid/Emergency Medicaid, Other) and onset time, defined as the time in days from first SARS-Cov-2 PCR positive admission in the Mount Sinai Health System to the individual patient’s admission.

### Statistical analysis

A two-step approach was undertaken in this analysis. First, we employed latent class analysis (LCA) to identify COVID-19 subphenotypes on admission without consideration of outcomes. The distribution and completeness of clinical and laboratory data was examined. As LCA allows for missingness, no data imputations were performed. Clinical and laboratory variables were placed into quintiles for LCA. We fit LCA models ranging from 2 to 10 subphenotype classes and then determined the best fitting model (i.e. the number of subphenotypes). Criteria for number of subphenotypes included: (1) consistent Akakie information criteria (cAIC) and adjusted Bayesian information criteria (aBIC), where lower values suggest better fit; (2) entropy, where higher values suggest better class separation; (3) likelihood ratio; and (4) number of participants per subphenotype, where models with adequate sample size in each class are optimal. Once the best fitting model and number of subphenotypes was identified, participants were assigned to the subphenotype for which they had the highest probability of correct assignment. These subphenotype assignments were then used as the independent variable for subsequent regression analyses.

Given that our COVID-19 outcomes of interest were common (occurring in more than 10% of the cohort), we employed bivariate and multivariable Poisson regression models with robust error variance to examine associations between COVID-19 subphenotype assignment and risk of mortality, ICU admission, and intubation, considered separately [32,33] using the R package *sandwich* [34,35]. Amongst survivors, we employed bivariate and multivariable generalized linear regression to examine associations between COVID-19 subphenotype and length of stay. Multivariable models adjusted for onset time, hospital, self-identified race/ethnicity and insurance provider. Finally, we explored whether the COVID-19 subphenotypes had a differential response to tocilizumaband convalescent plasma therapies through introduction of an interaction term and in treatment-stratified models.

Analyses were conducted in R software version 4.1.2. Latent class analyses were performed using the *poLCA* package [36,37].

## Results

A total of 4620 patients were admitted to the Mount Sinai Health System during the study period and met inclusion criteria (patient characteristics, Table 1). The largest percentage of patients in the cohort were admitted to Mount Sinai Hospital (34%, *n* = 1550), followed by Mount Sinai Queens (19%, *n* = 883), Mount Sinai Brooklyn (18%, *n* = 850) and Mount Sinai Morningside (18%, *n* = 838). Patients median age was 67 (IQR 55–78) years and 57% (*n* = 2629) were male. Twenty-eight percent (*n* = 1272), 27% (*n* = 1258), and 5% (*n* = 223) of patients self-identified as Hispanic, Black or Asian, respectively. Categories of co-morbidities were summed as described above, and the majority of the patients had zero (37%, *n* = 1715) or one (40%, *n* = 1,831) organ-specific comorbidity. Overall 29% (*n* = 1342) of patients died, 16% (*n* = 714) were intubated, and 21% (*n* = 972) were admitted to the ICU.

**Table 1.** Baseline characteristics (*N* = 4620).

	Number	Per cent
Age, years (median (range), IQR)	67 (18–110)	55, 78
Male	2629	56.9
Race/ethnicity (self-reported)		
Hispanic	1272	27.5
Black	1258	27.2
White	1094	23.7
Asian	223	4.8
Other	635	13.7
Missing	138	3.0
Insurance		
Medicaid	1076	23.2
Medicare/Private Insurance	3415	73.9
Other	97	2.1
Missing	32	0.7
Hospital		
MS Hospital	1550	33.5
MS Queens	883	19.1
MS Brooklyn	850	18.4
MS Morningside	838	18.1
MS West/Not specified	495	10.8
Missing	4	
Number of comorbidities^b^		
0	1715	37.1
1	1831	39.6
2	814	17.6
3	238	5.2
4	22	0.5
Time from onset of pandemic^a^, days (median (range), IQR)	28 (0–173)	20, 38
Outcomes		
Mortality	1342	29.0
Intubation	714	15.5
ICU admission	972	21.0
Length of stay (*N* = 3278 survivors), days (median (range), IQR)	6.2 (0.01–103)	3.1, 10.9

^a^Time in days from first SARS-Cov-2 PCR positive admission in the Mount Sinai Health System to the individual patient’s admission.

^b^Comorbidities included history of cancer, obesity, pulmonary disease (asthma, chronic obstructive pulmonary disease (COPD) and/or obstructive sleep apnea (OSA)), and cardiovascular disease (hypertension (HTN), coronary artery disease (CAD), congestive heart failure (CHF) and/or myocardial infarction (MI)).

### Latent class analysis

Overall, clinical and laboratory data completeness was high (Supplemental Table S1). Supplemental Figure S1 displays the LCA model fits, specifically cAIC, aBIC, entropy and likelihood ratio for models with 2–10 subphenotypes. Using these variables, we determined that the optimal fit was six subphenotypes.

**Figure 1.**
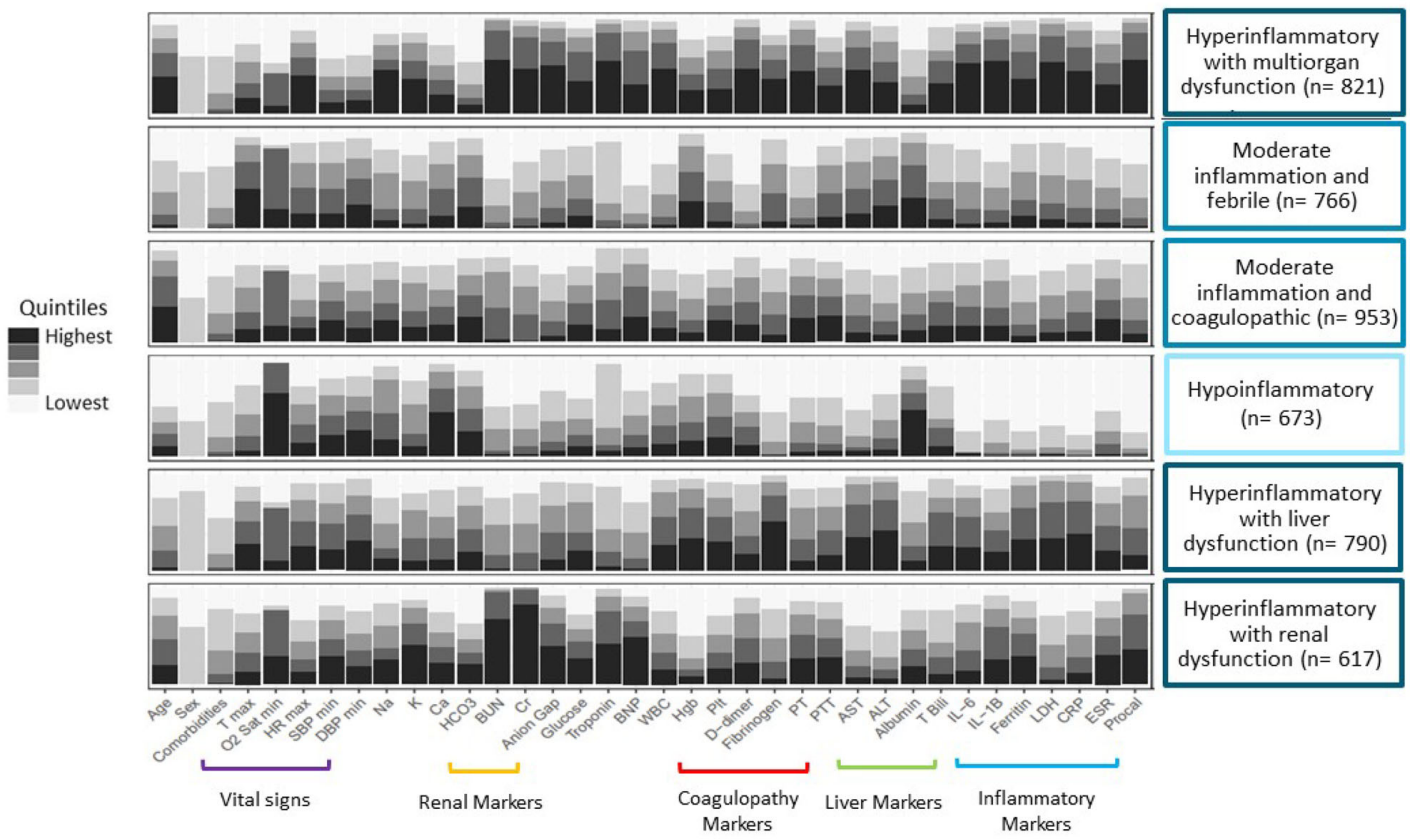
Distribution of clinical and laboratory variables on admission amongst the six COVID-19 subphenotypes. Clinical and laboratory variables on admission normalized and divided into quintiles. Latent class analysis identified six underlying COVID-19 subphenotypes which were notable for varying levels of inflammation, vital sign abnormalities and/or organ dysfunction. T max: maximum temperature; O2 Sat min: minimum oxygen saturation; HR max: maximum heart rate; SBP min: minimum systolic blood pressure; DBP min: minimum diastolic blood pressure; Na: sodium; K: potassium; Ca: calcium; HCO3: bicarbonate; BUN: blood urea nitrogen; Cr: creatinine; BNP: brain natriuretic peptide; WBC: white blood cell count; Hgb: hemoglobin; Plt: platelet count; PT: prothrombin time; PTT: partial thromboplastin time; AST: aspartate aminotransferase; ALT: alanine transaminase; T bili: total bilirubin; IL-6: interleukin 6; IL-1B: interleukin 1B; LDH: lactate dehydrogenase; CRP: c-reactive protein; ESR: erythrocyte sedimentation rate; Procal: procalcitonin.

The six subphenotypes appeared clinically distinct (Figure 1, Supplemental Table S1). We summarize these subphenotypes as: an elderly (median age 75.5 years, IQR 65, 85), hyperinflammatory with multiorgan dysfunction subphenotype (*n* = 821); a younger (median age 59 years, IQR 49, 66), febrile with moderate inflammation subphenotype (*n* = 766); an elderly (median age 77 years, IQR 67, 84), moderate inflammation and coagulopathic subphenotype (*n* = 953); a younger (median age 51 years, IQR 35, 69), predominantly female (64%) hypoinflammatory subphenotype (*n* = 673); a predominantly male (82.5%) hyperinflammatory with liver dysfunction subphenotype (*n* = 790); and a hyperinflammatory with renal dysfunction subphenotype (*n* = 617). The hypoinflammatory subphenotype had the fewest clinical and laboratory abnormalities thus was the referent class in subsequent analyses.

### Associations between COVID-19 subphenotype and mortality

**Figure 2.**
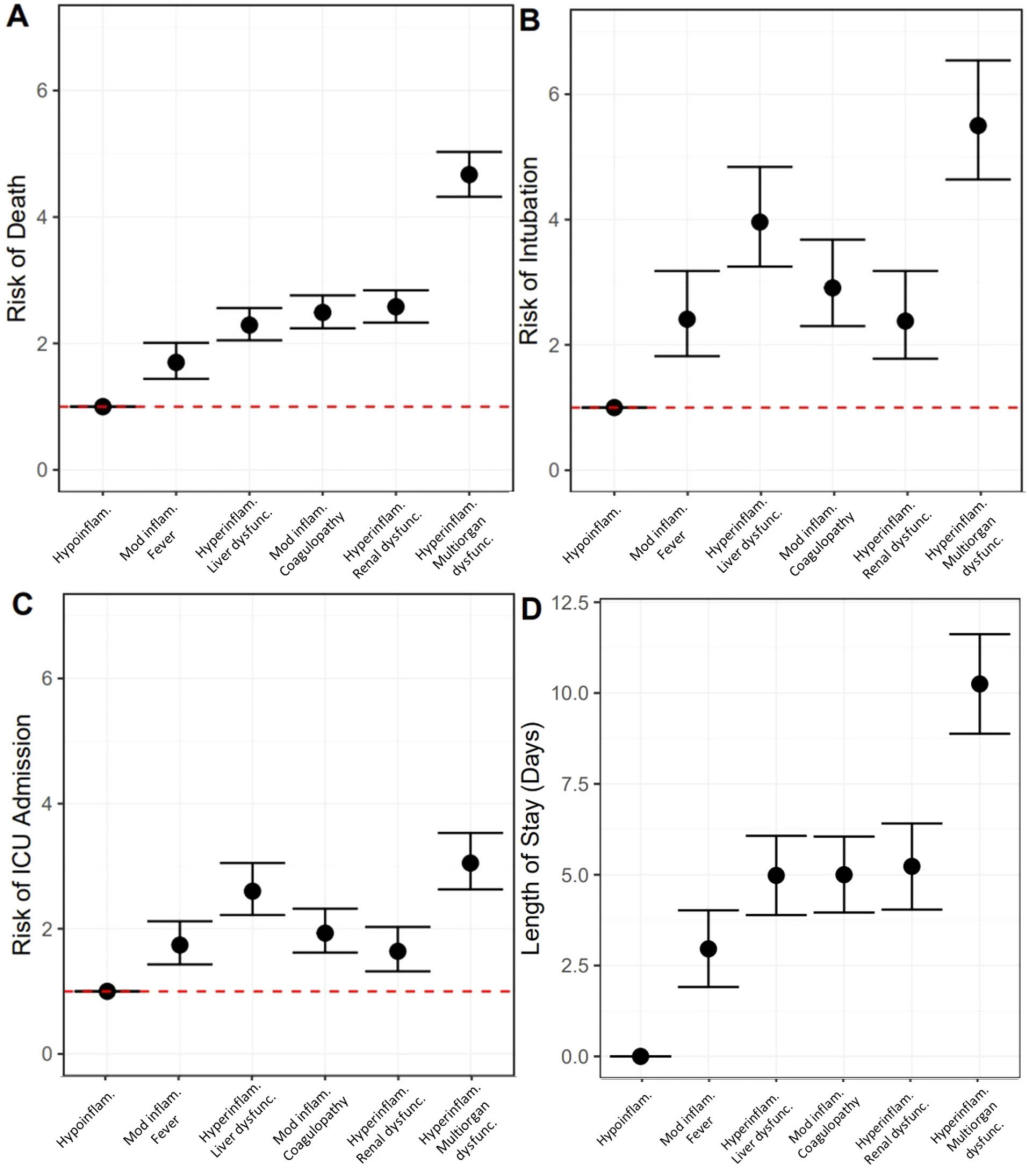
Associations between COVID-19 subphenotype and (A) mortality; (B) intubation; (C) intensive care unit (ICU) admission; and (D) length of stay amongst survivors. Robust Poisson regression to determine the relative risk of outcomes by subphenotype as compared to hypoinflammatory subphenotype where circles represent estimate and bars represent 95% confidence intervals. Multivariable model (shown here) adjusted for onset time, hospital, self-identified race/ethnicity and insurance provider. Hypoinflam.: Hypoinflammatory; Mod inflam. Fever: Moderate inflammation with fever; Hyperinflam. Liver dysfunc.: Hyperinflammatory with liver dysfunction; Mod inflam. Coagulopathy: Moderate inflammation with coagulopathy; Hyperinflam. Renal dysfunc: Hyperinflammatory with renal dysfunction; Hyperinflam. Multiorgan dysfunc.: Hyperinflammatory with multiorgan dysfunction.

Bivariate models suggest that, as compared to the hypoinflammatory subphenotype, all subphenotypes had increased risk of mortality (Supplemental Table S2). In multivariable models adjusting for onset time, hospital, self-identified race/ethnicity and insurance provider, all subphenotypes had increased risk of mortality as compared to the hypoinflammatory subphenotype (moderate inflammation and febrile RR 3.29, 95% CI 2.05, 5.28; hyperinflammation with liver dysfunction RR 6.85, 95% CI 4.37, 10.73; moderate inflammation and coagulopathic RR 8.09, 95% CI 5.19, 12.61; hyperinflammatory with renal dysfunction RR 8.78, 95% CI 5.61, 13.75; hyperinflammatory with multiorgan dysfunction RR 17.87, 95% CI 11.56, 27.63, Figure 2(A), complete model output Supplemental Table S2).

### Associations between COVID-19 subphenotype and intubation

In bivariate and multivariate models, all subphenotypes had increased risk of intubation as compared to the hypoinflammatory subphenotype (multivariable model: moderate inflammation and febrile RR 3.44, 95% CI 2.07, 5.74; hyperinflammation with liver dysfunction RR 7.49, 95% CI 4.60, 12.20; moderate inflammation and coagulopathic RR 4.72, 95% CI 2.87, 7.77; hyperinflammatory with renal dysfunction RR 3.30, 95% CI 1.95, 5.60; hyperinflammatory with multiorgan dysfunction RR 10.88, 95% CI 6.72, 17.62, Figure 2(B), complete model output Supplemental Table S3).

### Associations between COVID-19 subphenotype and ICU admission

In bivariate and multivariate models, all subphenotypes had increased risk of admission to the ICU as compared to the hypoinflammatory subphenotype (multivariable model: moderate inflammation and febrile RR 2.00, 95% CI 1.50, 2.67; hyperinflammation with liver dysfunction RR 3.31, 95% CI 2.52, 4.34; moderate inflammation and coagulopathic RR 2.25, 95% CI 1.71, 2.98; hyperinflammatory with renal dysfunction RR 1.84, 95% CI 1.35, 2.51; hyperinflammatory with multiorgan dysfunction RR 3.94, 95% CI 3.02, 5.15, Figure 2(C), complete model output Supplemental Table S4)

### Associations between COVID-19 subphenotype and length of stay

Amongst survivors (*n* = 3278), the median length of stay was 6.2 days (IQR 3.1, 10.9). Length of stay in days was longer in all subphenotypes as compared to the hypoinflammatory subphenotype in unadjusted models (multivariable model: moderate inflammation and febrile *β* = 2.95 days, 95% CI 1.88, 4.01; hyperinflammation with liver dysfunction *β* = 4.91 days, 95% CI 3.80, 6.02; moderate inflammation and coagulopathic *β* = 4.87 days, 95% CI 3.81, 5.93; hyperinflammatory with renal dysfunction *β* = 5.15 days, 95% CI 3.95, 6.34; hyperinflammatory with multiorgan dysfunction *β* = 10.19 days, 95% CI 8.79, 11.58, Figure 2(D), complete model output Supplemental Table S5).

### Exploratory analyses of COVID-19 therapies

**Figure 3.**
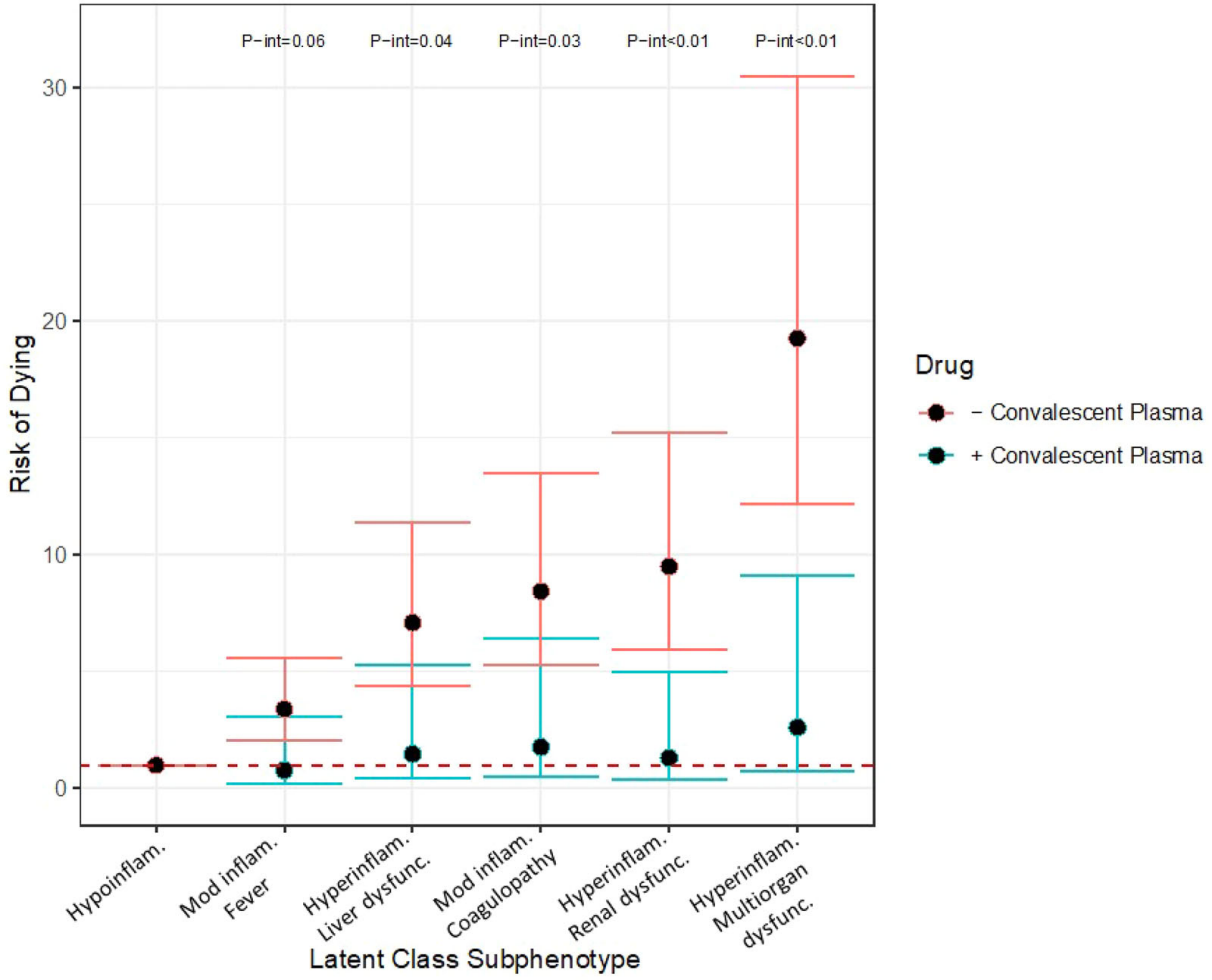
Risk of dying by subphenotype in those who did (blue bars) and did not (red bars) receive convalescent plasma. Robust Poisson regression models adjusted for onset time, hospital, self-identified race/ethnicity and insurance provider stratified by receiving convalescent plasma versus not. P-interaction terms generated by robust Poisson multivariable regression with introduction of an interaction term. P-interaction terms of less than 0.10 are shown. Hypoinflam.: Hypoinflammatory; Mod inflam. Fever: Moderate inflammation with fever; Hyperinflam. Liver dysfunc.: Hyperinflammatory with liver dysfunction; Mod inflam. Coagulopathy: Moderate inflammation with coagulopathy; Hyperinflam. Renal dysfunc: Hyperinflammatory with renal dysfunction; Hyperinflam. Multiorgan dysfunc.: Hyperinflammatory with multiorgan dysfunction. Number at risk per subphenotype (those who received convalescent plasma/those who did not): Hypoinflammatory (2/19), Moderate inflammation, fever (10/82), Hyperinflammatory, liver dysfunction (18/179), Moderate inflammation, coagulopathy (25/253), Hyperinflammatory, renal dysfunction (11/186), Hyperinflammatory multiorgan dysfunction (27/530).

Exploratory analyses to examine effect modification of the association between convalescent plasma (CP) and mortality by COVID-19 subphenotype suggested a differential effect by subphenotype. Within the cohort, *n* = 93 patients received CP. As compared to the hypoinflammatory subphenotype, the other subphenotypes demonstrated increased risk of dying in those who did not receive CP as compared to those who did [moderate inflammation with fever: received CP (*n* = 10) RR 0.85, 95% CI 0.22, 3.31 vs no CP (*n* = 82) RR 4.05, 95% CI 2.49, 6.59, p-int 0.06; hyperinflammatory with liver dysfunction: received CP (*n* = 18) RR 1.43, 95% CI 0.39, 5.24 vs no CP (*n* = 179) RR 8.59, 95% CI 5.42, 13.62, p-int 0.04; moderate inflammation with coagulopathy: received CP (*n* = 25) RR 1.64, 95% CI 0.46, 5.92 vs no CP (*n* = 253) RR 10.07, 95% CI 6.39, 15.87, p-int 0.03; hyperinflammatory with renal dysfunction: received CP (*n* = 11) RR 1.31, 95% CI 0.34, 5.00 vs no CP (*n* = 186) RR 11.29, 95% CI 7.14, 17.86, p-int <0.01; hyperinflammatory with multiorgan dysfunction: received CP (*n* = 27) RR 2.76, 95% CI 0.78, 9.76 vs no CP (*n* = 530) RR 23.96, 95% CI 15.34, 37.41, p-int <0.01, Figure 3].

**Figure 4.**
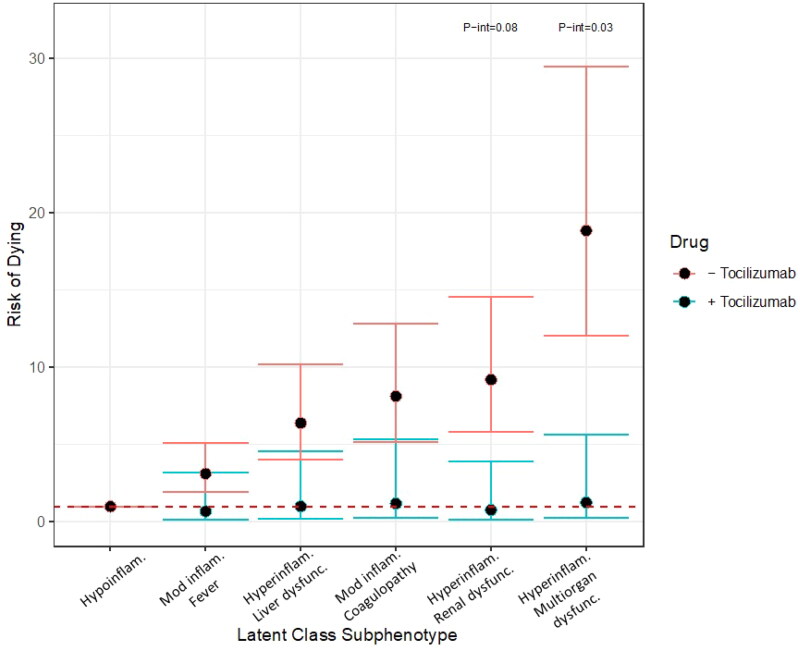
Risk of dying by subphenotype in those who did (blue bars) and did not (red bars) receive tocilizumab. Robust Poisson regression models adjusted for onset time, hospital, self-identified race/ethnicity and insurance provider stratified by receiving tocilizumab versus not. P-interaction terms generated by robust Poisson multivariable regression with introduction of an interaction term. P-interaction terms of less than 0.10 are shown. Hypoinflam.: Hypoinflammatory. Mod inflam. Fever: Moderate inflammation with fever; Hyperinflam. Liver dysfunc.: Hyperinflammatory with liver dysfunction; Mod inflam. Coagulopathy: Moderate inflammation with coagulopathy; Hyperinflam. Renal dysfunc: Hyperinflammatory with renal dysfunction; Hyperinflam. Multiorgan dysfunc.: Hyperinflammatory with multiorgan dysfunction. Number at risk per subphenotype (those who received tocilizumab/those who did not): Hypoinflammatory (1/20), Moderate inflammation, fever (14/78), Hyperinflammatory, liver dysfunction (37/160), Moderate inflammation, coagulopathy (26/252), Hyperinflammatory, renal dysfunction (6/191), Hyperinflammatory multiorgan dysfunction (34/523).

Within the cohort, *n* = 118 patients received tocilizumab. The associated risks for mortality in the hyperinflammatory with renal dysfunction and hyperinflammatory with multiorgan dysfunction, as compared to the hypoinflammatory subphenotype were higher amongst persons who did not received tocilizumab as compared to those who did (hyperinflammatory with renal dysfunction: received tocilizumab (*n* = 6) RR 1.29, 95% CI 0.23, 7.11 vs no tocilizumab (*n* = 191) RR 10.61, 95% CI 6.78, 16.60, p-int 0.08; hyperinflammatory with multiorgan dysfunction: received tocilizumab (*n* = 34) RR 2.00, 95% CI 0.40, 10.03 vs no tocilizumab (*n* = 523) RR 22.75, 95% CI 14.74, 35.13, p-int 0.03) (Figure 4).

## Discussion

Utilizing a cohort of 4620 COVID-19 patients during the Spring to Summer 2020 NYC surge, these data suggest that there are six distinct COVID-19 subphenotypes at the time of hospital admission. These subphenotypes have varying clinical courses, with differences in associated risk for mortality, intubation, ICU admission and LOS. Further, despite limited treatment options in the early pandemic, this work provides insight that COVID-19 subphenotypes may differentially respond to therapeutics, suggesting that further characterization of COVID-19 subphenotypes may be critical for future COVID-19 therapeutic trials and, ultimately, to guide therapy. This result expands on the currently published literature in two significant ways. It uses data at time of hospital admission, rather than trajectories while hospitalized or ICU admission, to provide the earliest possible timepoint to identify distinct subphenotypes [14,25,26]. Additionally, much of current literature focuses on strictly identifying subphenotypes [10,24,38]. This study goes beyond identification to begin to explore the role subphenotypes play in response to potential treatments.

Our admission dataset includes a number of serologic markers (IL-6, IL-1B, ferritin, LDH, ESR, CRP and procalcitonin) with variability across the cohort. The identified six subphenotypes differed predominantly in inflammatory profiles on admission, as defined by these serologic inflammatory markers. For example, median ferritin level on admission was 724 ng/ml (IQR 326, 1653) and varied amongst subphenotypes. The median ferritin in the hypoinflammatory subphenotype was 148.0 (IQR 56.8, 261.5) as compared to the hyperinflammatory subphenotypes (hyperinflammation with liver dysfunction median 1341.0 (IQR 770.2, 2465.2), hyperinflammation with renal dysfunction median 1004.0 (IQR 430.0, 2376.5) and hyperinflammation with multiorgan dysfunction subphenotype median 1441.5 (IQR 685.0, 2756.0)). We noted similarly separation of subphenotypes by IL-6, CRP and LDH in particular. While there is no single marker (serologic or otherwise) that has been identified to predict disease severity, associated organ involvement or outcome, multiple studies have demonstrated the role of IL-6, IL-8, CRP, LDH, procalcitonin and ferritin, in identifying patients at higher risk of poor outcomes [10,39–41]. Interestingly, the inflammatory markers are not uniformly elevated suggesting nuances in the inflammatory cascade and/or host response that need further investigation.

Organ dysfunction occurred predominantly in subphenotypes with the largest inflammatory derangements. For example, subphenotypes with hyperinflammation had multiorgan dysfunction or renal or liver failure. Notably, the liver failure group were predominantly younger (median age 61, IQR 52, 68) men which is not a group previously considered high risk [42]. Given the cross-sectional view of clinical and laboratory measures at admission we cannot temporally identify whether the inflammation directly led to the organ dysfunction or whether the inflammatory markers were elevated due to reduced renal or hepatic clearance [43]. It is interesting to note, however, that the two subphenotypes with moderately elevated inflammatory markers on average did not have renal or hepatic dysfunction. These data suggest that specific inflammatory markers in conjunction with markers of renal and hepatic injury may be used to identify patients at risk of clinical deterioration.

While the mortality risk observed for the most elderly group with multiorgan derangements is not surprising, it is important to note that this approach allowed us to identify subphenotypes that were at increased risk for mortality but may not previously have been considered to be at higher risk. For example, we identified a younger group (median age 59 years, IQR 49, 66) with moderate inflammation and febrile subphenotype that had 3.3 times the risk of death (RR 3.29, 95% CI 2.05 5.28), 3.4 times the risk of intubation (RR 3.44, 95% CI 2.07, 5.74) and 2 times the risk of ICU admission (RR 2.00, 95% CI 1.50, 2.67) in the multivariable model as compared to the hypoinflammatory subphenotype. On average, members of this group were previously healthy with no (*N* = 279, 36%) or one (*N* = 332, 43%) organ system comorbidity. It is critical that future studies focus on these populations to better understand the pathophysiologic mechanisms of increased risk, enable early identification and initiate appropriate treatments.

Further, these data suggest that identification of these subphenotypes at the time of hospital admission may be helpful in designing future COVID-19 therapeutic trials, guiding secondary analyses of existing COVID-19 randomized controlled treatment studies and, ultimately, in generating a patient-centered treatment algorithm. This work builds on prior analyses by Calfee et al. suggesting that ARDS subphenotypes differentially respond to therapeutic interventions in ARDS including fluid management strategies and use of statins [13,15,16].

Specifically, we find that the hyperinflammatory subphenotype with multiorgan dysfunction may differentially respond to tocilizumab and convalescent plasma therapies. Emerging evidence supports this concept and suggests that some treatments may be more efficacious in certain populations [39,44]. Current literature on the efficacy of tocilizumab and convalescent plasma have demonstrated conflicting results. Five large randomized trials examining the efficacy of tocilizumab, which enrolled patients with varying degrees of respiratory failure, found a mortality benefit in only two studies [45–49]. A recent meta-analysis of IL-6 antagonists, including tocilizumab, however did demonstrate a mortality benefit [50]. Similarly, studies of convalescent plasma have not shown mortality benefit but do suggest that early administration to mildly ill patients or with high titer plasma, may provide some benefit [51–55]. Our data support secondary analyses of completed randomized controlled trials to better understand if COVID-19 subphenotypes differentially respond to therapeutics. These analyses will be critical to inform patient-centered treatment algorithms.

Our study has several strengths. By leveraging the Mount Sinai electronic medical record data repository, we were able to evaluate a large, diverse sample of patients from multiple hospitals in New York City. We employed a data-driven method to analyze the heterogeneous population of COVID-19 patients and were able to identify underlying subphenotypes and demonstrate associations with mortality, ICU admission, intubation risk, and length of stay, key COVID-19 endpoints. Our exploratory analyses suggest that these subphenotypes may have a differential response to therapeutic treatments, which suggests that completed COVID-19 RCTs may benefit from secondary analyses of their datasets even if no effect in the overall cohort was found. Our employment of admission data to generate subphenotypes provides a platform for early identification of subphenotypes, which if replicated in other studies, suggests the potential for selecting therapeutic options based on identified differences early in the hospitalization.

We also acknowledge limitations. There is variability in duration of illness prior to presentation to the hospital that is not captured by this dataset. Patients may have presented at different phases of illness, which we know was true in New York City during the first wave of the pandemic as factors including patient volume and limited resources impacted decisions on when to present to the hospital or be admitted. Hospital capacity and available resources may have also impacted aggressiveness of treatment (e.g. palliative care) which we are unable to capture by electronic health records. We adjust for time since the onset of the pandemic to address this. An evaluation of the stability of the subphenotypes over time would lend additional evidence that the subphenotypes are distinct classes, regardless of duration of illness at presentation [56,57]. Exploratory analyses of associations with treatments are limited by potential biases as these data are retrospective. Access to these treatments was limited in Spring 2020. Certain treatments were not available at every hospital site and were restricted to patients with more severe illness rather than distributed in a randomized fashion, introducing selection bias. Criteria for use changed over time as more studies became available about these treatments, limiting generalizability of these analyses. Additionally, these data reflect the original strain of COVID, and were collected prior to the development of vaccines, which has impacted the clinical presentations and potentially altered the clinical subphenotypes.

In conclusion, these data find six distinct, clinically relevant, COVID-19 subphenotypes present on admission, which are associated with risk for mortality, intubation, ICU admission and LOS and suggest differential responses to tocilizumab and convalescent plasma. Future studies should validate these subphenotypes in other populations and health systems. Post hoc analyses of randomized control trials of tocilizumab, convalescent plasma and other therapeutics are warranted.

## Supplementary Material

Supplemental Material

## Data Availability

The deidentified data presented in this study is available upon request from the corresponding author.
