# Supplementary material for: COVID-19 subphenotypes at hospital admission are associated with mortality: a cross-sectional study
Source: Ann Med. 2022 Nov 29;55(1):12–23. doi: 10.1080/07853890.2022.2148733 (PMC10795648; doi:10.1080/07853890.2022.2148733)
Supplement: Supplemental Material [file IANN_A_2148733_SM5280.pdf]

Table S1. Admission characteristics including vital signs and lab values by COVID-19 subphenotype

	Hypoinflammatory (N = 673)	Mod. Inflam, fever (N = 766)	Hyperinflam, liver dysfunction (N = 790)	Mod. Inflam, coagulopathy (N = 953)	Hyperinflam, renal dysfunction (N = 617)	Hyperinflam, multiorgan dysfunction (N = 821)	Total (N = 4,620)	p-value [a]
**Age (years), N**	672	766	790	953	617	820	4,618	
Median (IQR)	51.0 (35.0, 69.0)	59.0 (49.0, 66.0)	61.0 (52.0, 68.0)	77.0 (67.0, 84.0)	70.0 (60.0, 79.0)	75.5 (65.0, 85.0)	67.0 (55.0, 78.0)	< 0.001
**Sex, N**	673	766	790	953	617	821	4,620	
Male, n (%)	242 (36.0%)	446 (58.2%)	652 (82.5%)	439 (46.1%)	363 (58.8%)	487 (59.3%)	2,629 (56.9%)	< 0.001
Female, n (%)	431 (64.0%)	320 (41.8%)	138 (17.5%)	514 (53.9%)	254 (41.2%)	334 (40.7%)	1,991 (43.1%)	
**Comorbidities, N**	673	766	790	953	617	821	4,620	
0, n (%)	311 (46.2%)	279 (36.4%)	357 (45.2%)	297 (31.2%)	134 (21.7%)	337 (41.0%)	1,715 (37.1%)	< 0.001
1, n (%)	234 (34.8%)	332 (43.3%)	308 (39.0%)	371 (38.9%)	269 (43.6%)	317 (38.6%)	1,831 (39.6%)	
2, n (%)	99 (14.7%)	125 (16.3%)	110 (13.9%)	198 (20.8%)	151 (24.5%)	131 (16.0%)	814 (17.6%)	
3, n (%)	27 (4.0%)	29 (3.8%)	15 (1.9%)	79 (8.3%)	56 (9.1%)	32 (3.9%)	238 (5.2%)	
4, n (%)	2 (0.3%)	1 (0.1%)	0 (0.0%)	8 (0.8%)	7 (1.1%)	4 (0.5%)	22 (0.5%)	
**T Max (°F), N**	673	766	790	953	616	820	4,618	
Median (IQR)	98.8 (98.3, 99.7)	100.9 (99.7, 102.1)	99.9 (98.8, 101.6)	99.2 (98.5, 100.3)	99.1 (98.3, 100.4)	99.0 (98.2, 100.4)	99.4 (98.5, 100.9)	< 0.001
**O2 Min (%), N**	670	766	789	953	616	820	4,614	
Median (IQR)	65 (56, 74)	66 (60, 73)	69 (62, 76)	61 (55, 68)	62 (54, 70)	56 (50, 66)	63 (56, 72)	< 0.001
**HR Max (bpm), N**	672	766	790	953	617	821	4,619	
Median (IQR)	98 (85, 109)	103 (94, 111)	106 (96, 118)	94 (84, 106)	94 (83, 106)	111 (95, 127)	100 (89, 114)	< 0.001
**SBP Min (mmHg), N**	673	766	789	952	617	820	4,617	
Median (IQR)	114 (102, 129)	114 (105, 125)	118 (107, 129)	114 (101, 129)	118 (103, 134)	102 (88, 118)	114 (101, 127)	< 0.001
**DBP Min (mmHg), N**	672	764	789	953	617	820	4,615	
Median (IQR)	65 (56, 74)	66 (60, 73)	69 (62, 76)	61 (55, 68)	62 (54, 70)	56 (50, 66)	63 (56, 72)	< 0.001
**Renal and hepatic markers**								
**ALT (IU/L), N**	582	740	754	905	582	787	4,350	
Median (IQR)	20.0 (14.0, 30.0)	35.0 (24.0, 55.0)	53.0 (34.0, 82.0)	21.0 (15.0, 32.0)	17.0 (12.0, 25.0)	39.0 (25.0, 72.0)	29.0 (18.0, 50.0)	< 0.001
**AST (IU/L), N**	554	707	725	844	539	720	4,089	
Median (IQR)	25.0 (19.0, 33.0)	44.0 (33.0, 62.0)	62.0 (45.0, 96.0)	35.0 (25.0, 51.0)	29.0 (21.0, 41.0)	73.0 (46.0, 117.0)	42.0 (28.0, 69.0)	< 0.001
**Albumin (g/dL), N**	573	743	755	909	586	795	4,361	
Median (IQR)	3.6 (3.1, 4.0)	3.5 (3.2, 3.7)	3.0 (2.7, 3.3)	3.0 (2.7, 3.4)	3.2 (2.7, 3.5)	2.9 (2.5, 3.3)	3.2 (2.8, 3.5)	< 0.001
**Tbili (mg/dL) , N**	572	744	757	908	584	795	4,360	
Median (IQR)	0.5 (0.3, 0.7)	0.5 (0.4, 0.7)	0.7 (0.5, 1.0)	0.6 (0.4, 0.8)	0.5 (0.4, 0.7)	0.7 (0.5, 1.1)	0.6 (0.4, 0.8)	< 0.001
**Sodium (mmol/L), N**	468	691	728	832	542	749	4,010	
Median (IQR)	138 (136, 140)	136 (133, 138)	135 (133, 138)	137 (134, 140)	137 (134, 141)	140 (135, 146)	137 (134, 140)	< 0.001
**Potassium (mmol/L), N**	595	736	769	919	598	782	4,399	
Median (IQR)	4.1 (3.8, 4.4)	4.0 (3.7, 4.4)	4.2 (3.8, 4.6)	4.2 (3.8, 4.6)	4.7 (4.1, 5.2)	4.5 (4.0, 5.1)	4.2 (3.8, 4.7)	< 0.001
**Calcium (mg/dL), N**	612	764	790	951	612	817	4,546	
Median (IQR)	8.9 (8.5, 9.3)	8.4 (8.1, 8.7)	8.3 (8.0, 8.7)	8.3 (7.9, 8.8)	8.3 (7.9, 8.9)	8.2 (7.8, 8.8)	8.4 (8.0, 8.8)	< 0.001
**HCO3 (mmol/L), N**	613	765	790	951	612	816	4,547	
Median (IQR)	23.4 (21.0, 26.0)	23.9 (21.8, 25.2)	23.0 (21.0, 25.0)	23.2 (21.0, 26.0)	21.0 (17.6, 25.0)	19.8 (16.3, 22.0)	22.7 (20.0, 25.0)	< 0.001
**BUN (mg/dL), N**	621	765	789	951	611	816	4,553	
Median (IQR)	12.0 (8.0, 16.0)	12.0 (9.0, 15.0)	15.0 (12.0, 21.0)	20.0 (15.0, 27.0)	52.0 (39.0, 71.0)	46.0 (30.0, 69.0)	19.0 (12.0, 36.0)	< 0.001
**Creatinine (mg/dL), N**	622	765	790	951	612	817	4,557	
Median (IQR)	0.77 (0.63, 0.95)	0.86 (0.70, 1.02)	0.90 (0.79, 1.12)	1.00 (0.80, 1.26)	3.30 (2.18, 6.70)	1.83 (1.29, 2.99)	1.05 (0.80, 1.66)	< 0.001
**Anion Gap, N**	612	765	790	951	610	816	4,544	
Median (IQR)	10.8 (9.0, 12.2)	11.7 (10.0, 13.0)	12.8 (11.0, 14.1)	11.0 (9.2, 13.0)	14.3 (12.0, 16.8)	15.1 (13.1, 18.7)	12.3 (10.3, 14.6)	< 0.001
**Glucose (mg/dL), N**	614	764	789	945	611	808	4,531	
Median (IQR)	103.0, (89.0, 126.0)	113.0, (102.0, 138.3)	122.0, (108.0, 163.0)	119.0, (100.0, 166.0)	122.0, (94.5, 200.5)	150.5, (116.0, 227.0)	120.0, (101.0, 169.0)	< 0.001
**Cardiac markers**								

For Peer Review Only

**Troponin (ng/mL), N**	480	684	729	860	551	768	4,072	
Median (IQR)	0.01 (0.01, 0.01)	0.01 (0.01, 0.01)	0.01 (0.01, 0.02)	0.02 (0.01, 0.05)	0.07 (0.03, 0.16)	0.11 (0.04, 0.29)	0.02 (0.01, 0.07)	< 0.001
**BNP (pg/mL), N**	242	480	563	646	453	637	3,021	
Median (IQR)	25.9 (10.0, 68.6)	10.9 (10.0, 23.5)	23.0 (10.7, 49.1)	103.2 (41.1, 268.7)	241.8 (45.6, 1056.7)	136.7 (52.8, 348.3)	50.3 (16.7, 185.5)	< 0.001
**Hematologic markers**								
**WBC (x10E3/uL), N**	666	764	790	951	616	818	4,605	
Median (IQR)	7.0 (5.13, 9.50)	5.90 (4.70, 7.35)	9.27 (7.30, 11.90)	6.90 (5.10, 9.75)	7.00 (5.20, 9.95)	11.40 (8.53, 15.30)	7.70 (5.60, 10.70)	< 0.001
**Hgb (g/dL), N**	667	765	790	951	616	820	4,609	
Median (IQR)	13.0 (11.8, 14.0)	13.8 (12.9, 14.9)	14.1 (13.1, 15.0)	12.1 (10.7, 13.4)	11.0 (9.6, 12.6)	13.0 (11.2, 14.6)	13.0 (11.5, 14.3)	< 0.001
**Platelets (x10E3/uL), N**	665	763	789	946	613	819	4,595	
Median (IQR)	213.0 (169.0, 269.0)	183.0 (149.0, 233.5)	243.0 (190.0, 311.0)	197.0 (147.0, 276.0)	190.0 (141. 0, 254.0)	222.0 (162.5, 292.5)	208.0 (159.0, 274.0)	< 0.001
**Ddimer (ug/mL), N**	385	583	659	688	423	677	3,415	
Median (IQR)	1.190 (0.560, 2.670)	0.750 (0.525, 1.180)	1.590 (1.040, 2.950)	1.825 (1.088, 3.235)	2.120 (1.330, 3.710)	3.780 (2.290, 10.570)	1.720 (0.910, 3.490)	< 0.001
**Fibrinogen (mg/dL), N**	248	300	359	407	236	388	1938	
Median (IQR)	452.0 (390. 0, 532.0)	626.0 (543.0, 694.3)	777.0 (684.0, 871.0)	575.0 (467.5, 668.0)	569.0 (480.8, 652.8)	691.5 (539.3, 817.3)	618.0 (496.0, 744.0)	< 0.001
**PT (seconds), N**	414	411	467	607	387	540	2,826	
Median (IQR)	13.4 (12.9, 14.1)	13.4 (12.9, 13.9)	14.1 (13.5, 14.9)	14.5 (13.6, 16.0)	14.6 (13.7, 16.1)	15.6 (14.3, 17.3)	14.1 (13.4, 15.4)	< 0.001
**PTT (seconds), N**	412	409	455	597	382	533	2,788	
Median (IQR)	28.8 (26.8, 31.3)	32.1 (29.5, 34.9)	32.4 (29.6, 35.7)	32.7 (29.1, 37.9)	33.0 (29.4, 38.3)	33.2 (28.8, 39.1)	32.0 (28.7, 36.3)	< 0.001
**Inflammatory markers**								
**IL-6 (pg/mL), N**	166	345	350	363	227	318	1,769	
Median (IQR)	16.6 (9.0, 30.7)	51.7 (34.5, 79.5)	96.3 (54.0, 151.0)	66.4 (37.5, 124.0)	69.6 (41.2, 119.9)	185.5 (82.5, 447.0)	70.7 (35.7, 137.8)	0.102
**IL-1B (pg/mL), N**	87	203	190	199	151	146	976	
Median (IQR)	0.2 (0.2, 0.4)	0.4 (0.2, 0.5)	0.5 (0.2, 0.8)	0.4 (0.2, 0.6)	0.6 (0.3, 0.9)	0.7 (0.4, 1.3)	0.4 (0.2, 0.7)	< 0.001
**Ferritin (ng/mL), N**	408	626	676	738	463	658	3,569	
Median (IQR)	148.0 (56.8, 261.5)	632.5 (383.0, 1091.7)	1341.0 (770.3, 2465.3)	416.5 (229.0, 766.5)	1004.0 (430.0, 2376.5)	1441.5 (685.0, 2756.0)	724.0 (326.0, 1653.0)	< 0.001
**LDH (U/L), N**	400	596	634	656	395	579	3260	
Median (IQR)	254.5 (214.0, 315.0)	392.0 (327.0, 467.3)	545.0 (458.0, 702.5)	369.5 (281.0, 466.5)	347.0 (282.0, 428.5)	651.0 (504.5, 877.0)	421.5 (315.0, 573.0)	< 0.001
**CRP (mg/L), N**	419	630	688	739	452	667	3,595	
Median	18.5 (7.6, 40.3)	86.1 (53.4, 130.4)	191.4 (140.1, 259.9)	90.5 (52.1, 157.2)	92.5 (45.3, 149.1)	205.0 (121.5, 277.1)	114.3 (54.0, 198.4)	< 0.001
**ESR (mm/hr), N**	169	367	422	427	263	418	2,066	
Median (IQR)	30.0 (12.0, 50.0)	45.0 (29.0, 66.0)	69.0 (46.0, 93.0)	64.0 (41.0, 90.5)	70.0 (45.5, 102.0)	73.0 (48.0, 103.0)	61.0 (37.0, 88.0)	< 0.001
**Procalcitonin (ng/mL), N**	372	608	666	687	425	627	3,385	
Median (IQR)	0.04 (0.02, 0.06)	0.09 (0.06, 0.15)	0.25 (0.15, 0.58)	0.12 (0.07, 0.26)	0.57 (0.28, 1.43)	1.02 (0.43, 3.28)	0.19 (0.08, 0.65)	< 0.001

Clinical and laboratory data from the first 24 hours of the patient's first hospitalization. Comorbidities = number of co-morbidities. T max= Temperature max. O2 min= minimum O2 saturation. HR max= maximum heart rate. SBP min = minimum systolic blood pressure. DBP min = minimum diastolic blood pressure. HCO3 = bicarbonate. BUN = blood urea nitrogen. BNP = brain natriuretic peptide. WBC = white blood cell count. Hgb= hemoglobin. PT = prothrombin time. PTT = partial thromboplastin time. AST = aspartate aminotransferase. ALT = alanine transaminase. T bili = total bilirubin. IL-6 = interleukin 6. IL-1B = interleukin 1B. LDH = lactate dehydrogenase. CRP = c-reactive protein. ESR = erythrocyte sedimentation rate.

a. ANOVA and chi-square tests examined differences across groups.

For Peer Review Only

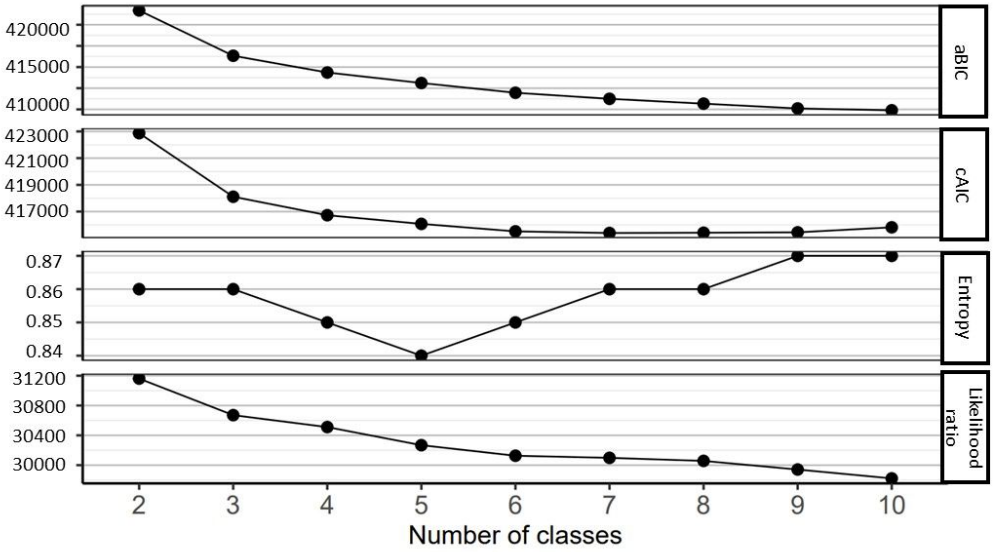

Figure S1. Latent class analysis (LCA) model fit for models with two through ten subphenotype classes. aBIC = adjusted Bayesian information criteria. cAIC = consistent Akakie information criteria.

For Peer Review Only

Table S2. Associations between COVID-19 subphenotypes and mortality

	Bivariate Model (n=4620)			Multivariable Model (n=4449)		
	RR	95% CI	p	RR	95% CI	p
Moderate inflammation, fever	3.85	2.42, 6.11	<0.01	3.29	2.05, 5.28	<0.01
Hyperinflammatory, liver dysfunction	7.99	5.16, 12.38	<0.01	6.85	4.37, 10.73	<0.01
Moderate inflammation, coagulopathy	9.35	6.07, 14.41	<0.01	8.09	5.19, 12.61	<0.01
Hyperinflammatory, renal dysfunction	10.23	6.61, 15.83	<0.01	8.78	5.61, 13.75	<0.01
Hyperinflammatory, multiorgan dysfunction	21.74	14.23, 33.21	<0.01	17.87	11.56, 27.63	<0.01
Onset Time	-	-	-	0.99	0.99, 0.99	<0.01
MS Brooklyn vs MSH	-	-	-	1.34	1.19, 1.51	<0.01
MS Queens vs. MSH	-	-	-	1.28	1.14, 1.44	<0.01
Morningside vs. MSH	-	-	-	1.07	0.94, 1.22	0.33
MS West vs. MSH	-	-	-	0.82	0.68, 0.99	0.04
Black vs White	-	-	-	0.84	0.75, 0.94	<0.01
Hispanic vs White	-	-	-	0.91	0.81, 1.03	0.13
Asian vs White	-	-	-	0.98	0.80, 1.19	0.81
Other vs White	-	-	-	1	0.88, 1.14	0.95
Medicaid vs. Private/Medicare	-	-	-	0.8	0.71,0.90	<0.01
Other Insurance vs Private/Medicare	-	-	-	0.84	0.59, 1.17	0.30

Robust Poisson regression models to determine risk of mortality per subphenotype as compared to the hypoinflammatory subphenotype. Multivariable model adjusted for time since pandemic onset, race/ethnicity, hospital of presentation, and insurance. MS= Mount Sinai, MSH=Mount Sinai Hospital

For Peer Review Only

Table S3. Associations between COVID-19 subphenotypes and intubation

	Bivariate Model (n=4620)			Multivariable Model (n=4449)		
	RR	95% CI	p	RR	95% CI	p
Moderate inflammation, fever	4.13	2.48, 6.91	<0.01	3.44	2.07, 5.74	<0.01
Hyperinflammatory, liver dysfunction	8.67	5.32, 14.12	<0.01	7.49	4.60, 12.20	<0.01
Moderate inflammation, coagulopathy	5.28	3.21, 8.67	<0.01	4.72	2.87, 7.77	<0.01
Hyperinflammatory, renal dysfunction	3.79	2.23, 6.42	<0.01	3.3	1.95, 5.60	<0.01
Hyperinflammatory, multiorgan dysfunction	12.44	7.70, 20.11	<0.01	10.88	6.72, 17.62	<0.01
Onset Time	-	-	-	0.99	0.99, 1.00	<0.01
MS Brooklyn vs MSH	-	-	-	0.99	0.82, 1.20	0.93
MS Queens vs. MSH	-	-	-	0.75	0.63, 0.91	<0.01
Morningside vs. MSH	-	-	-	0.69	0.56, 0.84	<0.01
MS West vs. MSH	-	-	-	0.5	0.37, 0.68	<0.01
Black vs White	-	-	-	0.99	0.81, 1.21	0.90
Hispanic vs White	-	-	-	1.25	1.02, 1.54	0.03
Asian vs White	-	-	-	1.18	0.85, 1.64	0.33
Other vs White	-	-	-	1.29	1.03, 1.61	0.03
Medicaid vs. Private/Medicare	-	-	-	0.96	0.81, 1.14	0.63
Other Insurance vs Private/Medicare	-	-	-	0.51	0.26, 1.00	0.05

Robust Poisson regression models to determine risk of intubation per subphenotype as compared to the hypoinflammatory subphenotype. Multivariable model adjusted for time since pandemic onset, race/ethnicity, hospital of presentation, and insurance. MS= Mount Sinai, MSH=Mount Sinai Hospital

For Peer Review Only

Table S4. Associations between COVID-19 subphenotypes and intensive care unit (ICU) Admission						
	Bivariate Model (n=4620)			Multivariable Model (n=4449)		
	RR	95% CI	p	RR	95% CI	p
Moderate inflammation, fever	2.01	1.51, 2.68	<0.01	2.00	1.50, 2.67	<0.01
Hyperinflammatory, liver dysfunction	3.26	2.5, 4.26	<0.01	3.31	2.52, 4.34	<0.01
Moderate inflammation, coagulopathy	2.24	1.70, 2.95	<0.01	2.25	1.71, 2.98	<0.01
Hyperinflammatory, renal dysfunction	1.74	1.28, 2.36	<0.01	1.84	1.35, 2.51	<0.01
Hyperinflammatory, multiorgan dysfunction	3.77	2.90, 4.90	<0.01	3.94	3.02, 5.15	<0.01
Onset Time	-	-	-	1.00	1.00, 1.00	0.60
MS Brooklyn vs MSH	-	-	-	0.78	0.65, 0.93	<0.01
MS Queens vs. MSH	-	-	-	0.65	0.55, 0.78	<0.01
Morningside vs. MSH	-	-	-	1.01	0.87, 1.18	0.86
MS West vs. MSH	-	-	-	0.98	0.81, 1.18	0.83
Black vs White	-	-	-	1.01	0.85, 1.19	0.92
Hispanic vs White	-	-	-	1.17	0.99, 1.39	0.07
Asian vs White	-	-	-	0.98	0.73, 1.32	0.91
Other vs White	-	-	-	1.17	0.97, 1.42	0.10
Medicaid vs. Private/Medicare	-	-	-	1.08	0.94, 1.24	0.26
Other Insurance vs Private/Medicare	-	-	-	0.43	0.22, 0.83	0.01

Robust Poisson regression models to determine risk of ICU admission per subphenotype as compared to the hypoinflammatory subphenotype. Multivariable model adjusted for time since pandemic onset, race/ethnicity, hospital of presentation, and insurance. MS= Mount Sinai, MSH=Mount Sinai Hospital

For Peer Review Only

Table S5. Associations between COVID-19 subphenotypes and length of stay amongst survivors						
	Bivariate Model (n=3278)			Multivariable Model (n=3159)		
	RR	95% CI	p	RR	95% CI	p
Moderate inflammation, fever	2.99	1.96, 4.03	<0.01	2.95	1.88, 4.01	<0.01
Hyperinflammatory, liver dysfunction	4.55	3.49, 5.62	<0.01	4.91	3.80, 6.02	<0.01
Moderate inflammation, coagulopathy	4.87	3.84, 5.90	<0.01	4.87	3.81, 5.93	<0.01
Hyperinflammatory, renal dysfunction	4.89	3.72, 6.06	<0.01	5.15	3.95, 6.34	<0.01
Hyperinflammatory, multiorgan dysfunction	9.80	8.43, 11.17	<0.01	10.19	8.79, 11.58	<0.01
Onset Time				-0.02	-0.03, -0.004	0.01
MS Brooklyn vs MSH				-2.37	-3.41, -1.33	<0.01
MS Queens vs. MSH				-4.77	-5.77, -3.77	<0.01
Morningside vs. MSH				-0.66	-1.61, 0.29	0.17
MS West vs. MSH				-1.09	-2.17, -0.01	0.05
Black vs White				0.31	-0.64, 1.27	0.52
Hispanic vs White				0.45	-0.52, 1.42	0.36
Asian vs White				1.75	0.10, 3.40	0.04
Other vs White				1.02	-0.14, 2.17	0.08
Medicaid vs. Private/Medicare				-0.29	-1.08, 0.50	0.48
Other Insurance vs Private/Medicare				-2.54	-4.75, -0.34	0.02

Robust Poisson regression models to determine risk of intubation per subphenotype as compared to the hypoinflammatory subphenotype. Multivariable model adjusted for time since pandemic onset, race/ethnicity, hospital of presentation, and insurance. MS= Mount Sinai, MSH=Mount Sinai Hospital

For Peer Review Only
